# Affirming our commitment to diversity, equity, and inclusion

**DOI:** 10.1016/j.patter.2025.101204

**Published:** 2025-03-06

**Authors:** Alejandra Alvarado, Wanying Wang, Andrew L. Hufton

**Affiliations:** Scientific editor, *Patterns*; Scientific editor, *Patterns*; Editor-in-chief, *Patterns*

## Main text

Today, we affirm *Patterns’* commitment to promoting diversity, equity, and inclusion (DEI) in science. Numerous research studies have demonstrated the many benefits of diverse research teams, including enhanced innovation, more accurate representation of the populations served, and improved decision-making (see, e.g., Swartz et al., 2019, *J Infect Dis.*; Márquez-Magaña, 2024, *Patterns*; and references therein). Diversity, equity, and inclusion are themselves crucial concepts in data science, where the biases of models and analyses need to be rigorously understood.

We therefore see the January 20^th^ executive order issued by President Donald Trump ending DEI programs in all US federal agencies as a major setback to efforts to build a more diverse, equitable, and inclusive scientific workforce. We worry that it poses a threat to the progress that has been made in recent years to address inequities faced by women, LGBTQ+ individuals, racial and ethnic minorities, and other underrepresented groups in science. These communities already face numerous challenges in their pursuit of STEM careers, including bias, discrimination, and a lack of role models and mentors.

As a result of Trump's executive order, the National Institutes of Health (NIH) and the National Science Foundation (NSF) have already terminated or paused grants and contracts supporting DEI initiates, and other agencies have halted DEI-related activities (Miller and Rabin, 2025, *NY Times*). Additionally, non-governmental science funders are facing pressure to implement changes; for example, the Howard Hughes Medical Institute suspended millions in initiatives dedicated to enhancing diversity in science (Kozlov, 2025, *Nature*). These terminations come in addition to major cuts to overhead funding that will place further pressure on research institutions inside and outside the US (Maniatis, 2025, *Cell*).

One member of our team was supported by a grant from the imperiled US Agency for International Development as part of an initiative designed to promote higher education worldwide and thus advance global development.

This support enabled me to complete a bachelor's research project at the University of Georgia. As a foreigner, they had no obligation to support someone like me, but they did. This opportunity was pivotal for my professional trajectory. It motivated me to stay in school and ultimately made me a stronger candidate for graduate school, which I continued to in the United States. – Alejandra Alvarado

We believe that responsible data science requires consideration of DEI issues, in particular so that the biases and fairness of models and methods can be quantified and understood. This is highly relevant, for example, to researchers aiming to build safe and reliable AI models. AI research and data science ultimately require a nuanced consideration of these issues so that researchers can better imbue future technologies with our shared values. Any attempt to censor or avoid these concepts will only hinder research in this area and could potentially lead to models or technologies that are more likely to cause harm.

While recognizing our limits as editors, we pledge to do what we can to protect and support the voices of our authors and to ensure that the papers published at *Patterns* are not edited in a way that would undermine the integrity of the work or erase important concepts such as gender, bias, diversity, or equity. If authors submitting to the journal have any concerns about external pressures to edit or censor their work, we invite them to reach out to us so that we may have a frank discussion about whether the paper can be considered at the journal and how best to manage or mitigate political pressures that could undermine the work.

Further, we commit ourselves to continuing to feature perspectives from researchers from diverse backgrounds and to give visibility to underrepresented voices. We invite our readers, for example, to explore the excellent essays from the 5^th^ Annual Rising Black Scientists Awards, the diverse scientists profiled as part Cell Press’ 50^th^ anniversary celebration, and the papers in the Cell Press collection on building inclusivity in science.

We can push back against these restrictive policies in our own domains by promoting the use of inclusive language, supporting scientists from underrepresented backgrounds, and advocating for equitable science. Ultimately, science thrives on collaboration and inclusion of all voices. Silencing of these perspectives will undoubtedly slow innovation and lead to technologies that fail to serve all.

